# Impacts of Rumen Degradable or Undegradable Protein Supplementation on Supplement Intake and Performance of Yearling Heifers and Cows Grazing Dryland Pastures

**DOI:** 10.3390/ani12233338

**Published:** 2022-11-29

**Authors:** Marley K. Manoukian, Timothy DelCurto, Janessa Kluth, T. J. Carlisle, Noah Davis, Makae Nack, Samuel A. Wyffels, Abe Scheaffer, Tom W. Geary, Megan L. Van Emon

**Affiliations:** 1 Department of Animal Range Sciences, Montana State University, Bozeman, MT 59717, USA; 2 SweetPro LLC, Walhalla, ND 58282, USA; 3 Fort Keogh Livestock and Range Research Laboratory, United States Department of Agriculture—Agriculture Research Service, Miles City, MT 59301, USA

**Keywords:** beef cattle, supplement intake behavior, self-fed supplement, rumen degradable protein, rumen undegradable protein

## Abstract

**Simple Summary:**

This study was conducted to evaluate intake and performance of beef cattle provided different protein supplements on pasture. Protein sources were rumen degradable protein (RDP) and rumen undegradable protein (RUP) supplied as pressed blocks provided in two time periods. Protein supplement was provided before and during the breeding season. Intake, intake behavior, and weight gain were compared between cattle offered either protein type. Typically, due to the RDP available during the summer grazing season, supplementation is not conducted. However, due to the RDP content of forages, RUP may be beneficial for grazing animals to provide a more direct protein source for the animal. Therefore, this study analyzed how effectively RUP or RDP supplementation was for grazing animals and found that protein type did impact intake behavior of both heifers and cows.

**Abstract:**

Angus and Red Angus-based yearling heifers (*n* = 40) and lactating cows (*n* = 51) were each used in a complete randomized design and stratified by weight and body condition score to one of two treatments: (1) pressed supplement block containing rumen undegradable protein (RUP) and (2) pressed supplement block containing rumen degradable protein (RDP). Heifer and cow supplement intake displayed (*p* < 0.01) a treatment × period interaction. The RUP heifers and RDP cows consumed more in Period 2 than Period 1, whereas RDP heifers and RUP cows consumed more in Period 1 than Period 2, respectively. Intake rate demonstrated (*p* < 0.01) a treatment effect for heifers, with RUP consuming supplement faster than the RDP treatment. Intake rate for cows demonstrated (*p* < 0.01) a treatment × period interaction with RUP cows in Period 1 having faster intakes than Period 2, and RDP cows having the inverse. Cow intake variation displayed (*p* < 0.01) a treatment × period interaction with RUP cows having more variation in Period 2, while RDP cows had less variation in intake in Period 2. In conclusion, RDP and RUP impacted intake behavior of cows and heifers but had minimal impacts on performance.

## 1. Introduction

Beef cattle production is important in the western United States, and because of the integral role replacement heifers play, their development is important to ranching systems. The arid environment of the western United States causes forages to have seasonal deficiencies and often be low in protein [1], which is why supplementing heifers to meet their growth requirements may be necessary. Furthermore, retaining these heifers within the cowherd and supporting them as they continue to grow as well as during lactation and gestation is a challenge and may also require supplemental protein. Producers who depend on forages must optimize cattle productivity while minimizing input costs. Self-fed salt-limited supplements are often used to offset seasonal nutrient deficiencies and increase both animal performance and forage intake [2].

Previous research in our lab has indicated that rumen degradable protein (RDP) improved NDF digestibility compared to rumen undegradable protein (RUP) and those cows on a self-fed RDP supplement ate more than their RUP counterparts [3]. The improvement of fiber digestibility may lead to improved forage use during times when low-quality or high-fiber forages are available. Although RUP did not improve fiber digestion, the protein may be more readily available to the animal and have direct impacts on growth potential. However, another study observed no improvement of fiber digestibility of low-quality forages in lambs fed RUP compared to those fed RDP [4].

Previous research has also suggested that postpartum intake of differing protein sources did not impact calf preweaning gains [5,6,7]. The lack of differences may be due to the lack of change in milk composition except fat on day 20 postpartum, which was greater in cows fed a RDP source compared to those cows fed a RUP source [7]. Similarly, Dhuyvetter et al. [5] did not observe differences in milk production in lactating cows fed either a 25 or 50% RUP source. These studies suggest that calf performance may not be influenced by postpartum protein sources.

However, it is important to consider supplement intake behavior when measuring the impacts of RUP vs. RDP supplementation. Intake and consumption of feed is critical to determine the amount of nutrients consumed and available to the animal [8]. Variation in individual supplement intake can potentially impact an individual animal’s nutrient status and subsequent performance [9]. Knowledge of this variation in intake and its impacts are needed to develop a cost-efficient program for developing heifers [9] and for appropriate supplementation of the cow herd. Based on previous data, we hypothesized that the RUP supplemented cows and heifers would have improved performance due to the potential of providing a direct protein source to the animal. Therefore, the objective of this study was to evaluate the differences between RUP and RDP supplementation on supplement intake behavior and animal performance in both cows and heifers.

## 2. Materials and Methods

Experimental procedures described herein were approved by the Agriculture Animal Care and Use Committee of Montana State University (#2020-AA05). All animals used in this study were provided by the Montana Agricultural Experiment Station. This study was conducted at Fort Ellis (45°39′36.5″ N, 110°58′10.9″ W) and the Bozeman Agriculture Research and Teaching (BART; 45°39′41.1″ N, 111°04′24.9″ W) farms at Montana State University in Bozeman, MT. The average precipitation is 469 mm and of that, snow represents 59.3%. The average temperature is 9.74 °C and there is an average of 113 growing season days.

To evaluate dietary treatment effects, Angus and Red Angus-based yearling heifers (*n* = 40) were used in a complete randomized design and were stratified by weight and body condition score. Similarly, to evaluate dietary treatments on lactating cows (*n* = 51), cows also used in a complete randomized design and stratified by age, weight and body condition score. For both the heifer and lactating cow trials, cattle were randomly assigned within stratum to one of two dietary treatments: (1) salt-limited, pressed supplement block containing rumen undegradable protein (*n* = 20 heifers; *n* = 31 cows; RUP; 114.0 kg/block; SweetPro LLC, Wallhalla, ND, USA), and (2) salt-limited, pressed supplement block containing rumen degradable protein (*n* = 20 heifers; *n* = 30 cows; RDP; 15.11 kg/block). The RUP supplement contained 70% RUP and 30% RDP and the RDP supplement contained 37% RUP and 63% RDP. Animals were housed on pasture from 4 June 2020 to 26 August 2020 for an 84-d supplement intake trial with supplement intake being recorded beginning on d 4. The 84-d trial was separated into two periods: Period (1) d 4–d 45 and Period (2) d 46–d 84.

Each animal was considered an experimental unit. Animals were equipped with an electronic identification tag (Allflex USA, Inc., Dallas-Ft. Worth, TX, USA) for recording individual intake of supplement, number and length of visits, and intake rate. One SmartFeed Pro trailer (C-Lock Inc., Rapid City, SD, USA) was housed with the heifers and a second trailer was housed with the two and three-year old cows to provide the treatments. The SmartFeed Pro units can limit animal access to the feed bunk, which allows multiple treatments within a single pasture. Each trailer has four feeding units, resulting in two feeding units to supply the RUP and two feeding units to supply the RDP treatments within each trailer. Supplement samples were collected weekly, composited, and sent to a commercial laboratory (Dairy One, Ithaca, NY, USA) for complete proximate analysis and mineral concentrations (Table 1). Supplements were provided ad libitum to cattle grazing improved dryland pastures, with a formulated average intake of 0.45 kg/head/d.

Body weight (BW) and body condition scores (BCS) were collected at the beginning (d 0) and end (d 84) of the trial following a 16 h shrink. Calf BW was collected at the beginning and end of the trial following a 16 h shrink. Animal body condition was judged independently by two observers using a 9-point scale (1 = extremely emaciated, 9 = extremely obese; [10]) with the same technicians measuring body condition at both timepoints (Files S1 and S2).

Individual pasture production was measured by clipping a 0.25 m^2^ plot at 5 sites prior to animals entering the pasture and following their exit from the pasture. In the heifer rotation, pastures 1–3 corresponded with Period 1 and pastures 4 and 5 corresponded with Period 2. In the cow rotation, Pastures 1 and 2 corresponded with Period 1 and pastures 3 and 4 corresponded with Period 2. All clipped pasture samples were composited by pasture and pasture enter/exit and sent to a commercial laboratory (Dairy One, Ithaca, NY, USA) for complete proximate analysis and mineral concentrations (Table 2 and Table 3).

For both lactating cow and heifer trials, the effects of protein type on animal BW, BCS, calf weight, and calf ADG were analyzed using an analysis of variance (ANOVA) with a generalized linear model including treatment as a fixed effect. Daily individual supplement intake (g/d and g/kg BW/d), total protein intake (g/d), total RDP intake (g/d), total RUP intake (g/d), intake rate (g/min), and the coefficient of variation (% CV) of daily supplement intake for both trials were analyzed using ANOVA with a generalized linear mixed model including treatment, period, and a treatment × period interaction as fixed effects. Individual animal was used as the random intercept to account autocorrelation of multiple measurements for each individual animal. Data were plotted and transformed if needed to satisfy assumptions of normality and homogeneity of variance. An alpha ≤ 0.05 was considered significant. Means were separated using the Tukey method when *p* < 0.05. All statistical analyses were performed in R [11].

## 3. Results

Heifer average daily supplement intake expressed as g/heifer and g/kg BW both displayed (*p* < 0.01; Table 4) a treatment × period interaction. Period daily intake in g/heifer (*p* < 0.01) for the RUP supplemented group was 22% greater in Period 2 compared to Period 1, while RDP supplemented group had 45% less intake in Period 2 than Period 1. Daily intake in g/kg BW increased (*p* < 0.01) 17.5% for the RUP group from Period 1 to Period 2 and decreased 49% in the RDP group from Period 1 to Period 2. Total protein, RDP, and RUP intake demonstrated treatment × period interactions (*p* < 0.01), with RUP fed heifers having greater total protein, RDP, and RUP intakes than their RUP counterparts. Intake rate demonstrated (*p* < 0.01) a treatment effect, with RUP heifers consuming supplement 86% faster compared to the RDP heifers. The CV for heifer intake tended (*p* = 0.10) to display a treatment × period interaction, as the RUP heifers tended to have more variation in Period 1, while RDP heifers tended to have more variation in Period 2. There were no differences (*p* ≥ 0.48; Table 5) between initial and final BW and BCS, or ADG in RUP vs. RDP supplemented heifers.

Cow average daily supplement intake expressed as g/cow and g/kg BW both displayed (*p* < 0.01; Table 6) a treatment × period interaction with RDP cows having greater intakes compared to the RUP cows in both periods. In terms of daily intake in g/cow, cows on the RUP treatment consumed (*p* < 0.01) 51% more supplement in Period 1, while cows on the RDP treatment consumed 80% more supplement during Period 2. Total protein, RDP, and RUP intake demonstrated treatment × period interactions (*p* < 0.01), with lactating cows fed RDP having greater intakes of total protein, RDP, and RUP than their RUP counterparts. Intake rate displayed (*p* < 0.01) a treatment × period interaction with RUP animals consuming supplement 20% faster during Period 1, while RDP animals consumed supplement 42% faster during Period 2. Intake CV also displayed (*p* < 0.01) a treatment × period interaction with RUP cows having 9% more variation in Period 2, while RDP cows had 30% less variation in intake in Period 2.

In terms of cow performance, there was no difference (*p* ≥ 0.71; Table 7) in cow initial and final BW or BCS. There was a significant difference (*p* < 0.01) in cow ADG in which RDP supplemented cows had greater ADG than RUP supplemented cows. Calf initial BW did not differ (*p* = 0.19) between treatments. There was a tendency for calves of RDP supplemented cows to have greater final BW (*p* = 0.09) and ADG (*p* = 0.10) than calves of RUP supplemented cows.

## 4. Discussion

Research regarding individual supplement intake for animals in rangeland and pasture settings have only been reported recently. Mixed aged cows with access to a salt-limited supplement pre-calving during the winter months had variation in individual supplement intake across different forage qualities and quantities, as well as animal age [12]. In the first year, supplement intake decreased as age increased. However, in the second year, the 2- and 3-year-old group consumed more supplement than the yearlings, 4–5, and 6–7-year-olds. Similarly, in the current study, the lactating cows consumed more supplement than the yearling heifers. This may be due to the naivete of the yearling heifers compared to the cows and that the cows had previous experience with the trailers. Additionally, Sowell et al. [13] noted that herd structure and dynamics may also play a role in supplemental feeding, with older, dominant cattle over consuming supplement compared to younger, less dominant cattle. Moreover, Wellnitz et al. [14] observed similar results with 5- and 8-year-old cows consuming more total feed than 2-year-old cows, regardless of pregnancy or lactation status. However, this was attributed to overall rumen capacity, which also may have played a role in the current study.

Both McClain et al. [15] and White et al. [16] reported supplement intake behavior with yearling heifers on the same paddocks as our study in years previous. This has provided additional information on supplement intake variation. Yearling heifers provided salt-limited protein supplements had greater intakes in Period 2, compared to Period 1 [16]. While the cows on our study showed similar supplement intake behavior in the RDP treatment, the yearling heifers on the RDP treatment were the opposite with greater supplement intakes in Period 1 than Period 2. However, the opposite occurred in the RUP treatment, with the cows having greater intake during Period 1 and the heifers having greater intake in Period 2. This may be due to the poor-quality pasture that the cows were located on during their third rotation, while the heifers remained on moderate quality pasture during this time frame. Additionally, due to the low-quality pasture (6.5% CP) the cows were consuming, additional RDP may have been required, which may have led to the increased intake during period 2 for the RDP cows and a reduction in intake in the RUP cows. Previous work has been done demonstrating that RDP supplementation when lambs were consuming low-quality forages improved dry matter, organic matter, and NDF digestibility [17]. This improvement in digestibility of low-quality forages may have led to increased supplement intake by lactating cows. In contrast, DelCurto et al. [18] did not observe improvements in forage quality selection or organic matter digestibility in cannulated steers supplemented with protein. However, only the RDP cows consumed greater than the target intake of 0.45 kg/hd/d, all other treatments did not consume the target amount of supplement. The supplement intake less than the target amount may be partially explained by pasture quality.

In addition to hardness as a supplement intake limiter in the current project, salt was also used to limit intake to try to achieve the target intake of 0.45 kg/hd/d. However, based on the current results and previous research by Schauer et al. [19], forage quality may play a larger role in supplement intake than salt. Schauer et al. [19] observed an increase in supplement intake as the grazing season progressed from June to October regardless of the intake limiter utilized, salt, anionic salts, or calcium hydroxide. Additionally, Reuter et al. [20] observed that increased salt inclusion (45%) for self-fed supplements, intake remained highly variable and resulted in less than the target intake of 1 kg/hd/d. Variation in supplement intake in the current study was similar to Reuter et al. [20], however, based on previous research, forage quality may have played a larger role in supplement intake variation compared to salt. Supplement intake variation also played a large role in the total protein, RDP, and RUP intake levels of both lactating cows and heifers. Due to the increased supplement intake of RUP heifers, total protein, RDP, and RUP intake was greater than RDP heifers, whereas the opposite was true for lactating cows. These intakes, however, had minimal impacts on the performance of the cows and heifers.

In terms of intake rate in a previous study, yearling heifers that were provided protein blocks all had decreased intake rates in Period 2 compared to Period 1 [15]. In the current study, RUP supplemented cows had decreased intake rates in Period 2, while RDP supplemented cows had increased rates. This further suggests that RDP may have been lacking in pasture rotation 3, leading to the drastic increase intake. Additionally, SmartFeed Pro trailers were always available, and animals did not have to travel long distances to consume supplement in the current study, which may have led to the increased intake of RDP by cows. However, the RUP heifers had increased intake rates in period 2, which may suggest the opposite were true in the heifer rotations, where RDP was sufficient, but RUP may have been lacking. The large differences in intake may have also led to increased variation between animals within treatment.

Yearling heifers provided salt-limited protein supplements had decreased variation in supplement intake in Period 2 compared to Period 1 [16], as did yearling heifers provided protein blocks [15]. Cows on the current study had mixed supplement intake behavior results, as RUP supplemented cows had increased variation in Period 2 compared to Period 1, while RDP supplemented cows had decreased variation in Period 2 compared to Period 1. Yearling heifers on the current study had differing results compared to previous research on the same pastures as RUP heifers had more variation in intake in Period 1, and RDP heifers had more variation in Period 2. In part, this may be due to differences in forage quality compared to the previous studies and between periods. Forage quality may play a key role in supplementation intake, however, previous work by Atkinson et al. [4,21] has suggested that an optimum ratio of RDP to RUP is needed to maintain rumen efficiency when feeding lambs low-quality forage [21]. In contrast, Bohnert et al. [22] observed improvements of NDF intake on a per kg of BW basis in lambs supplemented with RUP compared with RDP, and this led to increased NDF digestibility. Therefore, protein supplementation aids in fiber digestibility, which may lead to improved performance.

An increase in supplement intake variation can exist among animals that are unfamiliar with the supplement delivery equipment [23]. Yearling heifers on the current study were not familiar with the SmartFeed Pro trailers, while the lactating cows had been exposed to the SmartFeed Pro trailers in previous studies. This may explain the yearling heifers having greater variability in supplement intake than the cows in this study. Additionally, this may explain the increased intakes of the lactating cows. Additional research using the SmartFeed Pro trailers conducted by Williams et al. [24] suggested that the trailers do not reduce individual intake variability. Loose supplements may be easier for animals to consume, as yearling heifers grazing the same pastures as the current study had greater intakes when supplement was in a loose or pelleted form compared to blocks [15,16]. Additionally, block hardness is used in self-fed supplements to control intake [2], which may also explain the increased variation in intake, which has been previously reported by [25]. Therefore, unfamiliarity with pressed supplement blocks and how to efficiently consume them may also explain the variation in supplement intakes in the yearling heifers.

In a study comparing RDP and RUP supplementation to first calf heifers, RDP supplemented postpartum did not impact weight change [26], which was similar to the current study in both the cows and yearling heifers. However, ADG was greater for RDP cows compared to RUP cows in the current study. This may suggest that the RUP cows may have been deficient in degradable protein, which led to the RDP cows having more rumen available protein for microbial efficiency and digestion, leading to greater ADG. Additionally, Schauer et al. [27] that protein supplementation in general increased BW and BCS change in cows compared to non-supplemented counterparts. Sletmoen-Olson et al. [28] observed increased BW and BCS in RUP fed lactating cows compared to the control cows. In contrast, previous research conducted with lactating Brahma heifers and cows suggested that increased RUP did not influence BW or BCS change during the first 119 d post-calving [29]. Additionally, Bohnert et al. [22] did not observe differences in cow BCS or BW when supplemented with either RDP or RUP. This suggests that there may be a potential benefit to supplementing RUP to cows during early lactation when nutrient requirements are greatest.

Additionally, the suckling calf weights in the [26] study were not impacted by supplementation, but calf final BW and ADG tended to be greater in the calves from RDP cows compared to the calves from RUP cows in the current study. In contrast, Rusche et al. [30] observed an increase in calf ADG when cows were fed a high-escape protein source compared to calves from cows that were fed a low-escape protein source. Moreover, Dhuyvetter et al. [5], Rakestraw et al. [6], and Wiley et al. [7] did not observe any differences in calf pre-weaning gain due to dam protein source supplementation. Although milk quality or quantity was not measured in the current study, milk quality or quantity may have been impacted in the RDP cows, which lead to an increase in ADG of their calves. However, this was not observed by Dhuyvetter et al. [5] and Triplett et al. [29], and only fat content was greater in cows fed RDP and only on d 20 postpartum in a study conducted by Wiley et al. [7]. The tendency for increased calf ADG may be explained by the intake amount of the RDP treatment by cows, which was approximately 2 and 3× greater in periods 1 and 2, respectively, than the target intake of 0.45 kg/hd/d. However, this was not the case in Rusche et al. [30], where only lactose concentrations were increased in cows fed a high-escape protein source.

## 5. Conclusions

Our results suggest that self-fed, salt-limited supplements have high degrees of intake variation, including variation among animals, over periods of time, and between types of protein. Rumen degradable protein supplementation has the potential to increase cow ADG and calf ADG. However, this only impacted the cows, there was no difference in heifer performance parameters. Therefore, lactation may have influenced nutrient partitioning leading to improved ADG in the RDP fed cows. Intake amount and rate were impacted by protein source in the heifers with the RUP heifers having greater intake and eating faster, whereas the inverse was true for the cows. Regardless of protein type overall supplement intake played a large role in the amount of protein, RDP, and RUP consumed each day. This research will contribute to the efforts to improve strategic supplementation of beef heifers and cows.

## Figures and Tables

**Table 1:** Nutrient analysis of supplement offered ad libitum to yearling, and 2- and 3-year- old cows in Smart Feed Pro Trailers while grazing improved dryland pasture.

	RUP ^1^	RDP ^1^
DM, %	77.3	86.6
Chemical composition, % DM		
Crude protein	27.8	37.0
Acid detergent insoluble crude protein	2.5	1.2
Soluble protein	13	15
Degradable protein, % crude protein	30	63
Acid detergent fiber	6.4	5.5
Total digestible nutrients	57	60
Ca	1.98	2.77
P	2.04	1.91
Mg	2.96	0.8
K	1.84	2.5
Na	6.15	4.3
S	1.23	0.73
Chemical composition, mg/kg		
Fe	852	1060
Zn	1300	1530
Cu	848	222
Mn	1060	726
Mo	1.5	8.1

^1^ Protein type of rumen degradable protein (RDP) or rumen undegradable protein (RUP). The RDP supplement included plant protein by-products (primarily soybean meal), salt, molasses products, monocalcium phosphate, calcium carbonate, bentonite, magnesium oxide, zinc amino acid complex, zinc sulfate, manganese amino acid complex, manganese sulfate, copper amino acid complex, copper sulfate, vitamin E supplement, vitamin A supplement, vitamin D_3_ supplement, ethylenediamine dihydroiodide, sodium selenite, and cobalt carbonate. The RUP supplement contained corn distillers dried grains with solubles, corn condensed distillers solubles, salt, corn gluten meal, magnesium oxide, monocalcium phosphate, calcium carbonate, canola oil, potassium chloride, yeast culture, zinc sulfate, manganese sulfate, copper sulfate, zinc polysaccharide complex, zinc proteinate, manganese polysaccharide complex, manganese proteinate, copper polysaccharide complex, copper proteinate, dried Aspergillus Oryzae fermentation extract, liquid Aspergillus Niger fermentation product, cobalt carbonate, sodium selenite, ethylenediamine dihydroiodide, vitamin A acetate, vitamin D_3_ supplement, and vitamin E supplement.

**Table 2:** Heifer pasture (1–5) forage quality and production at the time of entry and exit at the Montana State University Fort Ellis research farm.


Rotation ^1^	1	2	3	4	5
Production, kg/ha					
Enter	1453.9	2906.8	2620.6	2497.3	1572.6
Exit	1280.4	1300.9	1526.1	870.5	1644.2
DM, %	90.7	90.8	91.7	91.3	92.9
Chemical composition, % DM					
Crude protein	14.8	9.0	7.3	8.7	6.4
Acid detergent fiber	35.7	36.0	38.3	38.1	40.8
Neutral detergent fiber	58.7	57.9	57.2	54.2	66.2
Total digestible nutrients	61.0	61.0	61.0	62.0	59.0
Ca	0.41	0.42	0.44	0.54	0.51
P	0.41	0.29	0.28	0.23	0.23
Mg	0.11	0.17	0.16	0.18	0.19
K	2.54	2.03	1.79	1.73	1.61
Na	0.011	0.013	0.008	0.006	0.006
Chemical composition, mg/kg					
Cu	16	15	16	12	15
Fe	172	139	132	96	82
Mn	51	70	105	85	73
Mo	4.9	1.7	2.1	3.2	3.9
Zn	29	19	15	18	15

^1^ Rotation 1–3 corresponded with Period 1 and rotation 4 and 5 corresponded with Period 2.

**Table 3:** Cow pastures (1–4) forage quality and production at the time of entry and exit at the Montana State University Fort Ellis research farm.


Rotation ^1^	1	2	3	4 ^2^
Production, kg/ha				
Enter	2683.3	3553.4	3203.1	4958.0
Exit	831.1	1388.8	1371.8	1982.9
DM, %	91.5	91.4	91.8	93.0
Chemical composition, % DM				
Crude protein	9.0	7.9	6.5	10.9
Acid detergent fiber	38.8	36.5	35.2	35.3
Neutral detergent fiber	59.9	61.3	56.5	58.0
Total digestible nutrients	60.0	60.0	61.0	61.0
Ca	0.37	0.33	0.43	0.52
P	0.26	0.23	0.17	0.28
Mg	0.12	0.11	0.14	0.18
K	1.97	1.58	1.80	2.50
Na	0.009	0.006	0.011	0.011
Chemical composition, mg/kg				
Cu	12	15	16	9
Fe	232	122	70	58
Mn	80	40	112	47
Mo	2.3	2.7	3.1	2.5
Zn	17	18	15	17

^1^ Rotation 1 and 2 corresponded with Period 1 and rotation 3 and 4 corresponded with Period 2. ^2^ The 4th rotation was at the Bozeman Agriculture Research and Teaching farm.

**Table 4:** Intake, intake rate, and % coefficient of variation (CV) data of yearling heifers supplemented with rumen undegradable or degradable protein while grazing improved dryland pastures.

.	Treatment ^1^			*p*-Value ^3^		
Item	RUP	RDP	SEM ^2^	Treatment	Period	Trt × Period
Intake, g/heifer/d				0.06	<0.01	<0.01
Period 1	286.31	151.87	50.82			
Period 2	349.00	83.44	51.06			
Total protein intake, g/heifer/d				0.26	<0.01	<0.01
Period 1	79.58	56.19	14.82			
Period 2	97.02	30.87	14.88			
Total RDP intake, g/heifer/d				0.10	<0.01	<0.01
Period 1	37.64	23.86	6.00			
Period 2	29.11	20.68	6.02			
Total RUP intake, g/heifer/d				<0.01	0.08	<0.01
Period 1	55.72	18.54	9.51			
Period 2	67.91	10.19	9.55			
Intake, g/kg BW/d ^4^				0.06	<0.01	<0.01
Period 1	0.80	0.43	0.14			
Period 2	0.94	0.22	0.14			
Intake rate, g/min	84.38	11.52	10.44	<0.01	0.57	0.29
CV, % ^5^	187.84	201.42	17.79	0.43	0.14	0.10
Period 1	204.20	176.35	24.76			
Period 2	172.79	230.05	25.72			

^1^ Protein type of rumen degradable protein (RDP) or rumen undegradable protein (RUP). ^2^ Pooled standard error of the means presented. ^3^ *p*-values of main effects of treatment, period, and treatment × period. ^4^ BW, body weight. ^5^ CV, % coefficient of variation of daily supplement intake.

**Table 5:** Performance parameters of yearling heifers supplemented with rumen undegradable or degradable protein while grazing improved dryland pastures.

	Treatment ^1^			
Item	RUP	RDP	SEM ^2^	*p*-Value
Initial body weight, kg	334.1	335.6	9.77	0.91
Initial body condition score	4.89	4.95	0.06	0.48
Final body weight, kg	449.6	446.8	8.65	0.82
Final body condition score	5.63	5.65	0.06	0.83
Average daily gain, kg/d	1.38	1.32	0.09	0.66

^1^ Protein type of rumen degradable protein (RDP) or rumen undegradable protein (RUP). ^2^ Pooled standard error of the means presented.

**Table 6:** Intake, intake rate, and % coefficient of variation (% CV) data of cows supplemented with rumen undegradable or degradable protein while grazing improved dryland pastures.

	Treatment ^1^			*p*-Value ^3^		
Item	RUP	RDP	SEM ^2^	Trt	Period	Trt × Period
Intake, g/cow/d				<0.01	<0.01	<0.01
Period 1	337.44	836.19	74.07			
Period 2	164.93	1503.02	75.21			
Total protein intake, g/cow/d				<0.01	<0.01	<0.01
Period 1	93.77	309.05	26.77			
Period 2	45.85	556.12	27.13			
Total RDP intake, g/cow/d				<0.01	<0.01	<0.01
Period 1	28.12	206.80	17.49			
Period 2	13.75	372.60	17.69			
Total RUP intake, g/cow/d				<0.01	<0.01	<0.01
Period 1	65.75	102.43	9.76			
Period 2	32.09	183.52	9.96			
Intake, g/kg BW/d ^4^				<0.01	<0.01	<0.01
Period 1	0.72	1.76	0.16			
Period 2	0.33	3.00	0.16			
Intake rate, g/min				0.10	<0.01	<0.01
Period 1	30.41	36.97	2.80			
Period 2	24.08	52.65	5.70			
CV, % ^5^				0.03	< 0.01	< 0.01
Period 1	136.79	112.14	8.07			
Period 2	149.19	78.37	7.37			

^1^ Protein type of rumen degradable protein (RDP) or rumen undegradable protein (RUP). ^2^ Pooled standard error of the means presented. ^3^ *p*-values of main effects of treatment (Trt), period, and treatment × period. ^4^ BW, body weight. ^5^ CV, % coefficient of variation of daily supplement intake.

**Table 7:** Performance parameters of cows supplemented with rumen undegradable or degradable protein while grazing improved dryland pastures and the calves.

	Treatment ^1^			
Item	RUP	RDP	SEM ^2^	*p*-Value
Initial body weight, kg	474.3	472.5	11.26	0.91
Initial body condition score	4.85	4.83	0.06	0.80
Final body weight, kg	521.2	535.2	11.67	0.40
Final body condition score	5.28	5.37	5.33	0.46
Average daily gain, kg/d	0.56	0.75	0.04	<0.01
Calf initial body weight, kg	125.4	133.2	4.12	0.19
Calf final body weight, kg	215.5	227.0	4.68	0.09
Calf average daily gain kg/d	1.07	1.12	0.02	0.10

^1^ Protein type of rumen degradable protein (RDP) or rumen undegradable protein (RUP). ^2^ Pooled standard error of the means presented.

## Data Availability

Data analyzed for this manuscript is located in the Intake and Performance Supplementary File folder.

## Supplementary Materials

The following supporting information can be downloaded at: https://www.mdpi.com/article/10.3390/ani12233338/s1. File S1: Intake Analysis and File S2: Body weight and condition.
